# Update: Characteristics of a Nationwide Outbreak of E-cigarette, or Vaping, Product Use–Associated Lung Injury — United States, August 2019–January 2020

**DOI:** 10.15585/mmwr.mm6903e2

**Published:** 2020-01-24

**Authors:** Vikram P. Krishnasamy, Benjamin D. Hallowell, Jean Y. Ko, Amy Board, Kathleen P. Hartnett, Phillip P. Salvatore, Melissa Danielson, Aaron Kite-Powell, Evelyn Twentyman, Lindsay Kim, Alissa Cyrus, Megan Wallace, Paul Melstrom, Brittani Haag, Brian A. King, Peter Briss, Christopher M. Jones, Lori A. Pollack, Sascha Ellington, Lung Injury Response Epidemiology/Surveillance Task Force, Lung Injury Response Epidemiology/Surveillance Task Force, Amena Abbas, Adebola Adebayo, Sukhshant Atti, Tegan Boehmer, Elizabeth Carter, Gyan Chandra, Lindsay Eckhaus, Janet Hamilton, Mia Israel, Zheng Li, Caitlin Loretan, Ruth Lynfield, Nisha Nataraj, Mary Pomeroy, Caroline Schrodt, Herschel Smith, Kimberly Thomas, Angela Werner

**Affiliations:** ^1^National Center for Injury Prevention and Control, CDC; ^2^Epidemic Intelligence Service, CDC; ^3^National Center for Immunization and Respiratory Diseases, CDC; ^4^National Center for Chronic Disease Prevention and Health Promotion, CDC; ^5^Center for Surveillance, Epidemiology, and Laboratory Services, CDC; ^6^National Center on Birth Defects and Developmental Disabilities, CDC; ^7^Office of Minority Health and Health Equity, CDC.; National Center for Chronic Disease Prevention and Health Promotion, CDC; National Center for Immunization and Respiratory Diseases, CDC; Agency For Toxic Substances and Disease Registry, CDC; National Center for Environmental Health, CDC; National Center for Environmental Health, CDC; National Center for Chronic Disease Prevention and Health Promotion, CDC; National Center for Chronic Disease Prevention and Health Promotion, CDC; Council of State and Territorial Epidemiologists; Council of State and Territorial Epidemiologists; Agency For Toxic Substances and Disease Registry, CDC; National Center for Immunization and Respiratory Diseases, CDC; Minnesota Department of Health; National Center for Injury Prevention and Control, CDC; National Center for Emerging and Zoonotic Infectious Diseases, CDC; National Center for Emerging and Zoonotic Infectious Diseases, CDC; National Center for Injury Prevention and Control, CDC; Center for Surveillance, Epidemiology, and Laboratory Services, CDC; National Center for Environmental Health, CDC.

## Abstract

Since August 2019, CDC, the Food and Drug Administration (FDA), state and local health departments, and public health and clinical stakeholders have been investigating a nationwide outbreak of e-cigarette, or vaping, product use–associated lung injury (EVALI) (*1*). This report updates patient demographic characteristics, self-reported substance use, and hospitalization dates for EVALI patients reported to CDC by states, as well as the distribution of emergency department (ED) visits related to e-cigarette, or vaping, products analyzed through the National Syndromic Surveillance Program (NSSP). As of January 14, 2020, a total of 2,668 hospitalized EVALI cases had been reported to CDC. Median patient age was 24 years, and 66% were male. Overall, 82% of EVALI patients reported using any tetrahydrocannabinol (THC)-containing e-cigarette, or vaping, product (including 33% with exclusive THC-containing product use), and 57% of EVALI patients reported using any nicotine-containing product (including 14% with exclusive nicotine-containing product use). Syndromic surveillance indicates that ED visits related to e-cigarette, or vaping, products continue to decline after sharply increasing in August 2019 and peaking in September 2019. Clinicians and public health practitioners should remain vigilant for new EVALI cases. CDC recommends that persons not use THC-containing e-cigarette, or vaping, products, especially those acquired from informal sources such as friends, family members, or from in-person or online dealers. Vitamin E acetate is strongly linked to the EVALI outbreak and should not be added to any e-cigarette, or vaping, products (*2*). However, evidence is not sufficient to rule out the contribution of other chemicals of concern, including chemicals in either THC- or non-THC–containing products, in some reported EVALI cases.

States and jurisdictions voluntarily report data on confirmed and probable hospitalized or deceased EVALI patients to CDC weekly using established case definitions* and data collection tools^†^ (*1*). Self-reported substances used in e-cigarette, or vaping, products were assessed among EVALI patients, including the percentage reporting any or exclusive THC-containing product use, any or exclusive nicotine-containing product use, and use of both THC- and nicotine-containing products. To assess trends in possible EVALI-related ED visits, CDC and health departments developed a query to assess exposure to e-cigarette, or vaping, products as a reason for an ED visit^§^ (*3**,**4*).

As of January 14, 2020, all 50 states, the District of Columbia, the U.S. Virgin Islands, and Puerto Rico had reported 2,668 hospitalized EVALI patients (Table). Overall, 66% of patients were male. The median patient age was 24 years (range = 13–85 years), and 76% were aged <35 years. Most EVALI patients were non-Hispanic white (73%), and 15% were Hispanic. Among 2,022 hospitalized patients with information on substances used, 1,650 (82%) reported using any THC-containing product, and 1,162 (57%) reported using any nicotine-containing product; 669 (33%) reported exclusive THC-containing product use, and 274 (14%) reported exclusive nicotine-containing product use.

**TABLE:** Demographic and product use characteristics among hospitalized patients with e-cigarette, or vaping, product use–associated lung injury (EVALI) reported to CDC — United States, August 2019–January 2020*

Characteristic (no. with available information)	No. (%)^†^ (N = 2,668)
**Sex (2,606)**	
Male	1,731 (66)
Female	875 (34)
**Median age, yrs (range)**	24 (13–85)
**Age group (yrs) (2,619)**	
13–17	404 (15)
18–24	979 (37)
25–34	631 (24)
35–44	335 (13)
45–64	223 (9)
≥65	47 (2)
**Race/Ethnicity^§^ (1,856)**	
White	1,360 (73)
Black	64 (3)
American Indian/Alaska Native	12 (1)
Asian/Native Hawaiian/Other Pacific Islander	38 (2)
Other	97 (5)
Hispanic	285 (15)
**Case status (2,668)**	
Confirmed	1,401 (53)
Probable	1,267 (47)
Substances used in e-cigarette, or vaping, products (2,022)^ ¶,^**	
Any THC-containing product	1,650 (82)
Any nicotine-containing product	1,162 (57)
Both THC- and nicotine-containing product use	834 (41)
Exclusive THC-containing product use	669 (33)
Exclusive nicotine-containing product use	274 (14)
No THC- or nicotine-containing product use reported	44 (2)

**Abbreviation:** THC = tetrahydrocannabinol.

* For cases reported to CDC as of January 14, 2020.

^†^ Percentages might not sum to 100% because of rounding.

^§^ These were mutually exclusive groups. Whites, blacks, American Indians/Alaska Natives, Asians/Native Hawaiians/Other Pacific Islanders, and Others were non-Hispanic. Hispanic persons could be of any race.

^¶^ Limited to persons who reported vaping or dabbing at least one substance in the past 3 months.

** In the 3 months preceding symptom onset.

The weekly number of hospital admissions for EVALI reported to CDC peaked at 215 during the week of September 15, 2019 (Figure 1). Since then, the number of cases reported each week has continued to steadily decline. NSSP data show that the number of possible EVALI-related ED visits sharply increased during August 11–September 8, 2019, by a mean of 26 visits per million each week (95% confidence interval [CI] = 18–33) (Figure 2). The weekly visit rate peaked at 116 per million during the week of September 8, 2019, then decreased by an average of approximately four per million weekly visits (95% CI = 4–5) to 35 per million during the week of January 5, 2020. This remains higher than the rate of 23 per million ED visits during the week of August 18, 2019.

**FIGURE 1:**
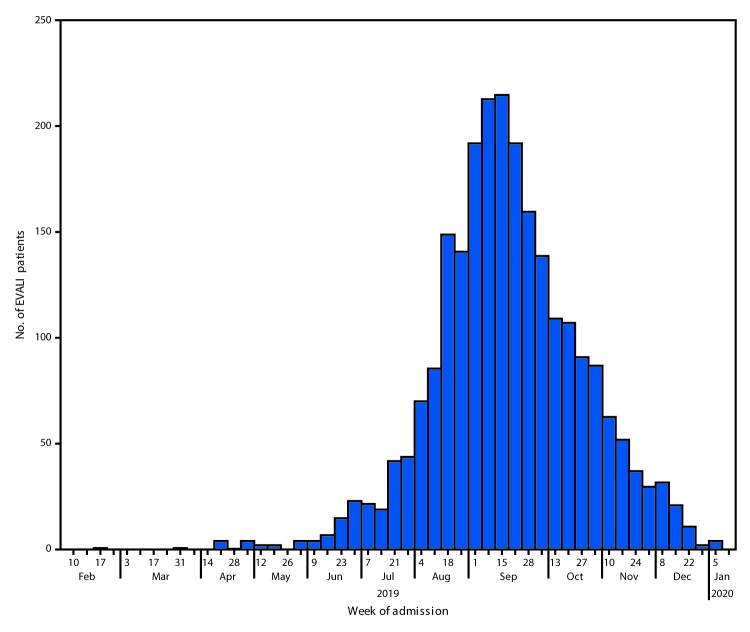
Number of patients (N = 2,398) with e-cigarette, or vaping, product use–associated lung injury (EVALI) by week of hospital admission — United States, February 10, 2019–January 14, 2020

## Discussion

As of January 14, 2020, all 50 states, the District of Columbia, the U.S. Virgin Islands, and Puerto Rico had reported EVALI patients. The majority of EVALI patients were non-Hispanic white, young adults, and male, similar to that reported previously (*1**,**5**,**6*). Most patients reported THC-containing product use. However, 14% reported exclusive use of nicotine-containing products.

Vitamin E acetate is strongly linked to THC-containing products used by EVALI patients (*2*). However, a minority of EVALI patients consistently report exclusive use of nicotine-containing products, which might be due to several factors. First, some patients might not accurately report, or know the content of, THC or other compounds in the products they have used (*2**,**7*). Second, some cases might be misclassified; for example, the high sensitivity of the EVALI case definition likely lowered specificity, leading to inclusion of some patients who do not have EVALI. Third, these patients might be accurately reporting exclusive use of nicotine-containing products (*7*). A previous report found a relatively low, but longstanding, background rate of ED visits associated with e-cigarette, or vaping, products predating the current outbreak, which could in part reflect one or more chemicals of concern in nicotine-containing products; however, this background rate could also reflect sporadic cases from the same products or substances that later contributed to the wider EVALI outbreak when they became more commonly used (*4*). The contributing cause or causes of EVALI for persons reporting exclusive use of nicotine-only products warrants further investigation.

Declines in the number of EVALI cases reported each week since mid-September 2019, and ED visits associated with e-cigarette, or vaping, products reported to NSSP, indicate that the outbreak peaked in September. Reasons for the decline might be multifactorial, including rapid public health action to increase public awareness of the risk associated with THC-containing e-cigarette, or vaping, product use, as well as actions by users to reduce this risk. Identification of the strong link between EVALI and vitamin E acetate, a diluent in THC-containing products, might have resulted in removal of vitamin E acetate from these products^¶^^,^** (*2**,**8**,**9*). Further, actions by enforcement agencies might have affected the supply of informally sourced THC-containing products (*8**,**10*). However, clinicians, public health practitioners, and the public should remain vigilant by taking steps to reduce risk, including efforts by clinicians to identify and treat EVALI patients.

The identification of EVALI as a new clinical syndrome highlights a need for further studies. Understanding the long-term health consequences of EVALI will require long-term patient follow-up. It is not known whether additives other than vitamin E acetate in e-cigarette, or vaping, products might cause similar lung injury. In addition, ongoing surveillance for lung injury associated with e-cigarette, or vaping, product use needs to continue to detect possible increases in lung injury if new additives (e.g., a harmful diluent other than vitamin E acetate) are added to these products in the future. Syndromic surveillance helped demonstrate that EVALI was a new clinical syndrome, with ED visits sharply increasing in August 2019 and declining after peaking in September 2019 (*4*).

The findings in this report are subject to at least three limitations in addition to those already discussed related to ascertainment of the product type used. First, data related to product use were missing for 24% of patients, and many EVALI patients were not interviewed because of loss to follow-up, refusal to be interviewed, or lack of resources to conduct interviews. Any of these factors might limit the generalizability of these findings to other EVALI patients. Second, the exposure query in NSSP might have been affected by public and clinical awareness of the outbreak, which increased the likelihood that e-cigarette, or vaping, products would be mentioned in stated reasons for ED visits. Finally, NSSP coverage is not uniform across or within states, and health care facilities contributing data change over time as new facilities are added to the system or removed when they close.

Based on data obtained in the investigation of EVALI since August 2019, CDC recommends that persons not use THC-containing e-cigarette, or vaping, products, particularly those from informal sources such as friends, family members, or from in-person or online dealers.^††^ Vitamin E acetate is strongly linked to the EVALI outbreak; it has been detected in product samples tested by FDA and state laboratories and in lung fluid samples from patients tested by CDC from geographically diverse states (*2*,*8**,**9*). Vitamin E acetate should not be added to any e-cigarette, or vaping, products. In addition, any substances not intended by the manufacturer should not be added to e-cigarette, or vaping, products, including to products purchased through retail establishments. However, evidence is not sufficient to rule out the contribution of other chemicals of concern, including chemicals in either THC- or non-THC–containing products, in some reported EVALI cases. Adults using e-cigarette, or vaping, products as an alternative to cigarettes should not go back to smoking; they should weigh all available information and consider using FDA-approved cessation medications.^§§^ They should contact their health care provider if they need help quitting tobacco products, including e-cigarettes, and if they have concerns about EVALI. Adults who do not currently use tobacco products should not start using e-cigarette, or vaping, products. Finally, e-cigarette, or vaping, products should never be used by youths, young adults, or pregnant women.

**Summary**

**What is currently known about this topic?**

Nationwide, 82% of patients hospitalized with e-cigarette or vaping, product use–associated lung injury (EVALI) reported tetrahydrocannabinol (THC)-containing product use. Vitamin E acetate, an additive to THC-containing e-cigarette, or vaping, products, is strongly linked to the EVALI outbreak.

**What is added by this report?**

The number of EVALI cases reported to CDC peaked during the week of September 15, 2019; the weekly number of hospitalized patients has since steadily declined.

**What are the implications for public health practice?**

Clinicians and public health practitioners should remain vigilant for EVALI cases. CDC recommends that persons not use THC-containing e-cigarette, or vaping, products, particularly from informal sources. Evidence is not sufficient to rule out the contribution of other chemicals of concern, including chemicals in either THC- or non-THC–containing products, in some reported EVALI cases.

**FIGURE 2:**
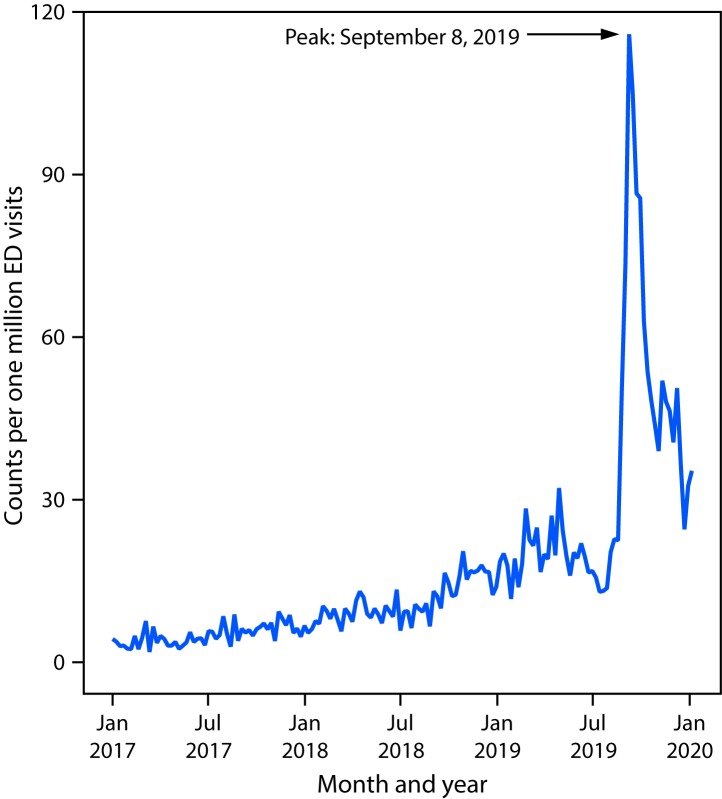
Emergency department (ED) visits with e-cigarette, or vaping, product use in the reason for visit (chief complaint)* — National Syndromic Surveillance Program, United States, January 1, 2017–January 11, 2020 * Excludes injuries unrelated to e-cigarette, or vaping, product use–associated lung injury (e.g., device explosions and accidental ingestion of e-liquid) but does not exclude potentially related syndromes such as acute intoxication from tetrahydrocannabinol or nicotine poisoning.

## Footnotes

* https://www.cdc.gov/tobacco/basic_information/e-cigarettes/assets/2019-Lung-Injury-Surveillance-Case-Definition-508.pdf.

† https://www.cdc.gov/tobacco/basic_information/e-cigarettes/severe-lung-disease/healthcare-providers/pdfs/National-Case-Report-Form-v01.pdf.

§ NSSP records free-text comments about the reason for ED visit, discharge diagnosis codes, and patient demographic characteristics from approximately 70% of ED visits nationwide.

¶ https://www.detroitnews.com/story/news/local/michigan/2019/12/17/michigan-recalls-marijuana-vaping-products-vitamin-e-acetate/2679157001/.

** https://www.cnn.com/2019/12/24/health/black-market-vapes/index.html.

†† https://www.cdc.gov/tobacco/basic_information/e-cigarettes/severe-lung-disease.html.

§§ https://www.cdc.gov/tobacco/campaign/tips/quit-smoking/index.html?s_cid.

## References

[1] Moritz ED, Zapata LB, Lekiachvili A, ; Lung Injury Response Epidemiology/Surveillance Group. Update: characteristics of patients in a national outbreak of e-cigarette, or vaping, product use–associated lung injuries—United States, October 2019. MMWR Morb Mortal Wkly Rep 2019;68:985–9. https://www.cdc.gov/mmwr/volumes/68/wr/mm6843e1.htm?s_cid=mm6843e1_w 31671085 10.15585/mmwr.mm6843e1 PMC6822806

[2] Blount BC, Karwowski MP, Shields PG, ; Lung Injury Response Laboratory Working Group. Vitamin E acetate in bronchoalveolar-lavage fluid associated with EVALI. N Engl J Med 2019;NEJMoa1916433. 10.1056/NEJMoa1916433 PMC7032996 31860793

[3] CDC. National Syndromic Surveillance Program (NSSP): what is syndromic surveillance? Atlanta, GA: US Department of Health and Human Services, CDC; 2019. https://www.cdc.gov/nssp/overview.html

[4] Hartnett KP, Kite-Powell A, Patel MT, Syndromic surveillance for e-cigarette, or vaping, product use–associated lung injury. N Engl J Med 2019. https://www.nejm.org/doi/10.1056/NEJMsr1915313 10.1056/NEJMsr1915313 PMC10613510 31860794

[5] Ellington S, Salvatore PP, Ko J, ; Lung Injury Response Epidemiology/Surveillance Task Force. Update: product, substance-use, and demographic characteristics of hospitalized patients in a nationwide outbreak of e-cigarette, or vaping, product use–associated lung injury—United States, August 2019–January 2020. MMWR Morb Mortal Wkly Rep 2020;68. 10.15585/mmwr.mm6902e2 10.15585/mmwr.mm6902e2 31945038 PMC6973348

[6] Chatham-Stephens K, Roguski K, Jang Y, ; Lung Injury Response Epidemiology/Surveillance Task Force; Lung Injury Response Clinical Task Force. Characteristics of hospitalized and nonhospitalized patients in a nationwide outbreak of e-cigarette, or vaping, product use–associated lung injury—United States, November 2019. MMWR Morb Mortal Wkly Rep 2019;68:1076–80. 10.15585/mmwr.mm6846e1 31751326 PMC6871898

[7] Ghinai I, Navon L, Gunn JKL, Characteristics of persons who report using only nicotine-containing products among interviewed patients with e-cigarette, or vaping, product use–associated lung injury —Illinois, August–December 2019. MMWR Morb Mortal Wkly Rep 2020;69. 10.15585/mmwr.mm6903e1 PMC7367041 31971930

[8] Taylor J, Wiens T, Peterson J, Characteristics of e-cigarette, or vaping, products used by patients with associated lung injury and products seized by law enforcement—Minnesota, 2018 and 2019. MMWR Morb Mortal Wkly Rep 2019;68:1096–1100. 10.15585/mmwr.mm6847e1 PMC6881051 31774740

[9] Food and Drug Administration. Lung illnesses associated with use of vaping products. Silver Spring, MD: US Department of Health and Human Services, Food and Drug Administration; 2019. https://www.fda.gov/news-events/public-health-focus/lung-illnesses-associated-use-vaping-products

[10] Food and Drug Administration. FDA, DEA seize 44 websites advertising sale of illicit THC vaping cartridges to US consumers as part of Operation Vapor Lock. Silver Spring, MD: US Department of Health and Human services, Food and Drug Administration; 2019. https://www.fda.gov/news-events/press-announcements/fda-dea-seize-44-websites-advertising-sale-illicit-thc-vaping-cartridges-us-consumers-part-operation

